# 

**Published:** 1861-12

**Authors:** 


### CHICAGO MEDICAL JOURNAL.

**Terms, $2.00 per Annum.**

### CONTENTS OF THE DECEMBER NUMBER.

ORIGINAL COMMUNICATIONS.

PAGE.

"Enucleation of the Eye." By E. L. Holmes, M. D., of Chicago, Ills..... 691

An Interesting Case of Pregnancy. By M. M. Eaton, M. D., Peoria....... 696

A Case of Inflammation of the Labia Pudenda. By J. B. Hoag, M. D.... 697

BOOK NOTICES.

The Principles and Practice of Obstetrics. By Gunning S. Bedford, A. M., M. D., Prof. of Obstetrics, the Diseases of Women and Children, and Clinical Obstetrics in the University of New York, etc., etc...... 700

Placenta Prævia—Its History and Treatment. By William Read, M. D., Member of the Massachusetts Medical Society, etc., etc.......... 705

Medical Jurisprudence. By Afred Swaine Taylor, M. D., F. R. V. S. Fifth American, from the 7th and Revised London Edition, etc., etc..... 708

EDITORIAL.

Editorial and Miscellaneous........................................ 710

SELECTED.

Moral Insanity in Relation to Criminal Acts. By L. Parigot, M. D., Late Commissioner in Lunacy and Chief Physician of the Colony of Gheel, Belgium, etc., etc........................................ 726

Exsection of the Hip-Joint........................................ 736

Rise and Progress of Clinical Teaching. Extract from an Address by J. L. Ludlow, M. D., Philadelphia, at the opening of a new Clinical Lecture Room........................................ 739

Pirogoff's Operation........................................ 746

Penalty of Overwork........................................ 747

The Age of Uterine Disease........................................ 748

Amount of Army Rations per Month........................................ 751

